# Collaborative treatment of late-life depression in primary care (GermanIMPACT): study protocol of a cluster-randomized controlled trial

**DOI:** 10.1186/1745-6215-15-351

**Published:** 2014-09-06

**Authors:** Iris Wernher, Frederike Bjerregaard, Iris Tinsel, Christiane Bleich, Sigrid Boczor, Thomas Kloppe, Martin Scherer, Martin Härter, Wilhelm Niebling, Hans-Helmut König, Michael Hüll

**Affiliations:** Institute on Aging, School of Community Health, Portland State University, PO Box 751, Portland, OR 97207 USA; Division of Psychiatry and Psychotherapy, Psychotherapy and Health Services Research, University Medical Center Freiburg, Hauptstr. 5, 79104 Freiburg, Germany; Department of Medicine, Division of General Practice, University Medical Center Freiburg, Elsässerstr. 2m, 79110 Freiburg, Germany; Department of Medical Psychology, University Medical Center Hamburg-Eppendorf, Martinistr. 52, 20246 Hamburg, Germany; Department of Primary Medical Care, University Medical Center Hamburg-Eppendorf, Martinistr. 52, 20246 Hamburg, Germany; Department of Health Economics and Health Services Research, Center for Health Economics, University Medical Center Hamburg-Eppendorf, Martinistr. 52, 20246 Hamburg, Germany; Center for Psychiatry, Emmendingen and University of Freiburg, Medical School, Lehenerstr. 88, 79106 Freiburg, Germany

**Keywords:** Collaborative care, Late-life depression, Stepped care

## Abstract

**Background:**

Depression is not a normal side effect of aging, however it is one of the most prevalent mental health issues in later life, imposing a tremendous burden on patients, their families, and the healthcare system. We describe the experimental implementation of a collaborative, stepped-care model for the treatment of late-life depression (GermanIMPACT trial) in the German primary care context. GermanIMPACT was developed as an adaptation of a successful and widely used American model. The aim of the study is to evaluate the model’s applicability to the German primary care setting and its cost-effectiveness.

**Methods/Design:**

The study will be conducted as a cluster-randomized controlled trial comparing the development of depressive symptoms in primary care patients who either receive treatment as usual (control arm) or treatment according to the GermanIMPACT model (intervention arm). In two German cities (Freiburg and Hamburg), a total of 60 general practice offices will be selected and randomized. Each general practice office will be asked to enroll five patients into the trial who are 60 years of age or older and who show moderate depressive symptoms in the scope of a diagnosed depressive episode, recurrent depressive disorder, or dysthymia. General practices in the control arm will provide treatment as usual; general practices in the intervention arm will work closely with a specially trained care manager and a supervising mental health specialist. Evidence-based elements of the treatment plan manual include patient education, identification and integration of positive activities into the daily routine, relapse prevention, and training of problem-solving techniques as needed. The intervention period per patient will be one year. Data will be collected at baseline, 6, and 12 months. Primary outcome is the patient-reported change of depressive symptoms (Patient Health Questionnaire, PHQ-9). Secondary outcomes include measures of quality of life, anxiety, depression-related behavior, problem-solving skills, resilience, and an overall economic evaluation of the program.

**Discussion:**

The GermanIMPACT trial will provide evidence about the effectiveness, feasibility, and cost-effectiveness of collaborative stepped care in treating late-life depression in German primary care. Positive results will be a first step toward integrating specialized depression care managers into the primary care setting.

**Trial registration:**

German Clinical Trials Register: DRKS00003589 (September 2012).

## Background

### Late-life depression

Depression, including major depression and less severe forms with clinically relevant depressive symptoms, is one of the most prevalent mental disorders in older adults and is considered a serious public health problem. International studies show that major depression affects up to 9.3% of individuals aged 75 years or older; less severe forms occur with a frequency of up to 37.4% [1, 2]. Depression, especially in combination with morbidity, disability, and social disconnectedness, is one of the key risk factors for suicide in later life [3]. To date, there are only a few studies on incidence rates of depression in older adults. Although findings vary considerably depending on diagnostic criteria and study method, some results indicate that incident rates in people between 60 and 70 years of age are equal to or lower than those found in the younger age groups [4]. A recent epidemiological field study suggested that the incidence and prevalence of depression rise again upon reaching the age of 70, and that cohort effects may further aggravate this phenomenon [5].

In primary care, the diagnostic situation is complicated by the fact that late-life depression often manifests as a mix of somatic conditions and cognitive impairment [6, 7] and often does not present with complaints regarding mood [4]. Hence, it can be assumed that a high number of older adults suffering from depression remain undiagnosed [4, 8].

Moreover, many people suffering from depression are poorly educated about the treatability of mood disorders. Yet another factor to be taken into account is the stigma associated with mental illness, especially for the older generation and within the German historical context. As a result of self-stigmatization, many older adults may consider depression a sensitive topic and be less likely to report their symptoms or consult a psychiatrist or psychotherapist [9]. Besides these psychological factors, the lack of sufficient regional mental health services and reduced mobility in older age may contribute to the low number of older patients treated by mental health specialists. While older adults suffering from major depression in Germany tend to see their general practitioner twice as often as they would without depression, they only have an average of 0.7 consultations with a psychiatrist or 1.3 consultations with a psychotherapist over a period of 12 months [10]. Given a recommended minimum of four psychiatric consultations over the course of 12 months for successful treatment, this means that only 17.5% of older patients receive a depression-specific intervention.

### Collaborative depression care

Cross-national research provides ample evidence for the advantage of structured collaborative depression care (CDC) approaches over traditional therapeutic interventions in primary care [11, 12]. Translational research has encouraged widespread implementation of CDC in diverse practice settings [13]. In the United States, several CDC programs have been evaluated and applied in practice for more than a decade. Although more research is needed regarding the cost-effectiveness of the collaborative care approach, recent studies are yielding promising results [14].

One core feature of CDC in primary care is low-threshold case management provided by trained nursing staff or counseling professions, such as social workers [12, 15]. In addition, the involvement of a supervising mental health specialist (MHS) has proven to be a crucial and discretely effective element in the interdisciplinary treatment of depression [13, 16–18].

In 2002, Unützer *et al*. [19] conducted an internationally recognized and frequently cited randomized controlled trial on their CDC model (IMPACT: Improving Mood - Promoting Access to Collaborative Treatment). Since then, the model has successfully been applied in many American primary care institutions. The IMPACT treatment plan includes a care manager (CM) and a supervising MHS. It is designed as a stepped-care model in which the intervention is tailored to the patients’ individual needs, allowing for optimal treatment results and minimal costs. Moreover, IMPACT has proven feasible and effective not only in the treatment of depression, but of other psychiatric conditions as well, namely anxiety and panic disorders [20].

### IMPACT: key agents and core elements

**Figure 1:**
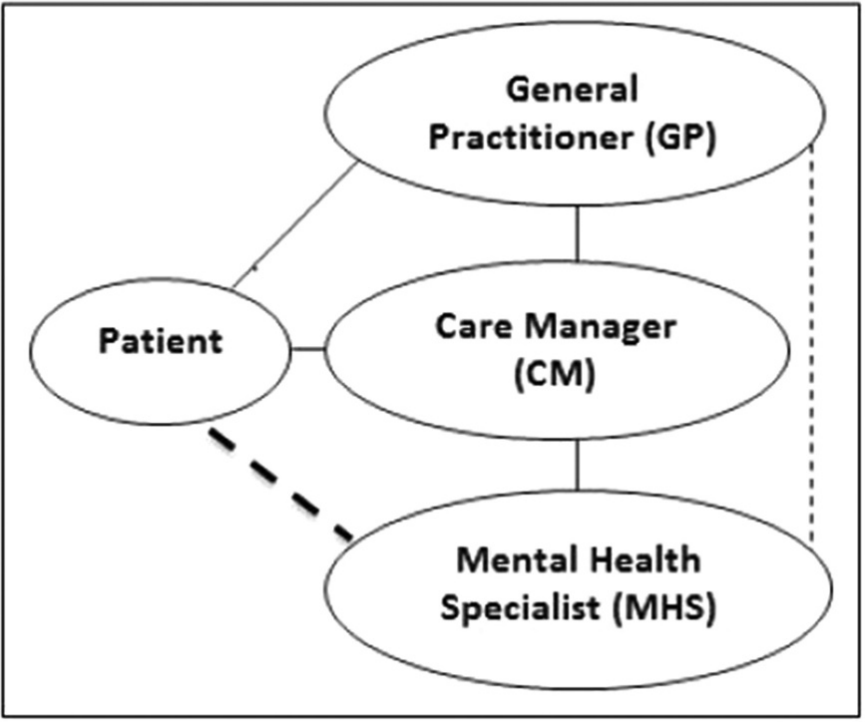
**IMPACT treatment triad (solid line: regular contact; broken line: contact as needed).**

The following three professional key agents (providers) form the IMPACT treatment triad are depicted in Figure 1.

The first agent in the interdisciplinary triad is the patient’s primary care provider, typically a general practitioner (GP) in an individual office for the German context. The GP diagnoses or confirms the diagnosis of depression and initiates treatment. He or she communicates with the CM on a regular basis in order to exchange information about the patient’s progress and to discuss adequate treatment adaptation as needed.

As central figure in the intervention process, a trained nurse or social worker in the role of the CM supports the treatment initiated by the GP by means of proactive and continuous follow-up with the patient. Intervention techniques provided by the CM typically include patient education (regarding symptoms, course of disorder, medication, side effects and so on), identification and integration of positive activities into the daily routine, relapse prevention, and training in problem-solving techniques as needed. These therapeutic elements have been proven to be effective in the treatment of depression and are recommended on high evidence levels by international clinical guidelines [21] (based on the procedure used by the Center of Evidence Based Medicine, all therapeutic elements hold the highest evidence degree [22]).

The third agent in the IMPACT treatment triad is a consulting and supervising MHS, that is, a psychiatrist or psychotherapist. The MHS supervises the intervention through regular meetings with the CM and provides professional guidance in difficult cases. He or she can be contacted by the GP regarding medical treatment options and is available in case of an emergency. In exceptional cases and after prior consultation with the CM, the GP can refer a patient directly to the MHS, for example, if the patient does not respond to the treatment.

Patient education and counseling provided by the CM focuses on the modification of cognitive appraisal and behavior, a powerful tool in the psychotherapeutic treatment of depression. Problem-solving techniques (PST) as an element of cognitive behavioral therapy are integrated into the treatment plan according to the patient’s individual symptom remission or lack thereof. The method has proven successful in numerous depression trials and can easily be acquired by a wide variety of professionals working in the healthcare field [23].

### Study aim and objectives

In recent years, clinical researchers around the globe have shown an increased interest in the applicability of collaborative care models, such as IMPACT, in different cultural settings and with different patient populations [15, 24]. In Europe, the model has been studied extensively in the Netherlands and the United Kingdom [25–30]. Until now, there have been only a few studies in Germany. Existing studies have not involved an MHS as a core intervention component, for instance, in the form of psychiatric supervision [31, 32].

The GermanIMPACT trial aims at broadening the scope of generalizability and the body of evidence for the adaptability of the IMPACT model to the German primary care context. Mental health research indicates that sustainable intervention programs need to be adapted carefully to existing systems and practices [13]. Thus, one of the key challenges was to create a concept compatible with the specific context of primary care in Germany without affecting the fidelity of the model, especially the core philosophy and elements.

Positive results in terms of symptom remission in collaboratively treated depressive patients will reinforce the cross-national applicability of the IMPACT core concept. In addition, such results will underline the impact of behavioral interventions in depression care and their successful implementation outside the specialized setting of high-frequency psychotherapy. The results will serve the goal of improving the treatment of late-life depression by paving the way for evidence-based intervention models in primary care. Proving the efficacy of the collaborative approach and its cost-effectiveness will be the first step towards its implementation into the German healthcare system.

## Methods/Design

### Setting

GermanIMPACT is a cooperation of the university medical centers of Freiburg and Hamburg-Eppendorf, Germany. Both centers serve as study sites, each with 30 participating GPs. The GPs will be recruited through the centers’ respective general practice departments. The Freiburg medical center is the coordinating center and is in charge of the management of data collected in Freiburg. The Hamburg medical center is responsible for the statistical analysis, assessments related to health economics, and the management of data collected in Hamburg. At each site, two CMs with a background in nursing are appointed and trained. Their offices are based at the university medical centers. This allows for easy access to the supervising MHSs who are part of the GermanIMPACT team and clinical staff at the medical centers.

Due to the geographic dispersion of German GP offices and the lack of additional practice space, most follow-up contacts will be conducted over the telephone. Research has shown that telephone interventions are a common and effective tool in depression care [12, 17]. Mandatory regular visits requiring traveling on the part of the patients would most likely be perceived as cumbersome and lead to dropouts. Additional face-to-face sessions will be scheduled only if considered necessary by the treatment triad.

### Sample size

The remission of depressive symptoms as measured by the Patient Health Questionnaire PHQ-9 at 12 months’ follow-up will be the primary outcome measure of the study. A randomized controlled trial investigating the effectiveness of the IMPACT program in the United States yielded a remission rate of 25.0% in the intervention group and 8.3% in the control group [19]. Assuming these remission rates in our trial along with a conventional value for the type I error rate (α = 0.05), a two-sided test on inequality of proportions requires 85 patients per arm (170 in total) to detect this effect with a power of 0.8 (type II error rate β = 0.2). Calculations were done with STATA 12.1 [33]. However, in order to account for the hierarchical structure of the collected data due to the cluster-randomized design, it must be considered that patients treated by the same GP are likely to be more similar in terms of treatment response than patients treated by different GPs. Assuming a fixed number of included GPs (30 per arm, 60 in total) and an amount of variance in the outcome explained by differences between physicians rather than between patients (intraclass correlation coefficient of 10%), the factor of the necessary increase in sample size (so-called ‘design effect’) due to the clustered design yields 1.46, increasing the required sample size to a total of 250 patients. This is the number needed for an appropriately powered per-protocol analysis (only completers are analyzed). To compensate for a conservatively estimated 15% loss to follow-up [19, 31], each participating GP will be instructed to include 5 patients in the study, amounting to a total of 300 patients allocated to the trial (150 per arm). The primary analysis will be performed in the intention-to-treat population, that is, all randomized patients will be included. In the intention-to-treat analysis, the higher number of patients is likely to be compensated for by a potential dilution of treatment effects, so that the power will be approximately the same as described for the per-protocol analysis.

### Recruitment of participants and randomization

#### Recruitment of GPs

**Figure 2:**
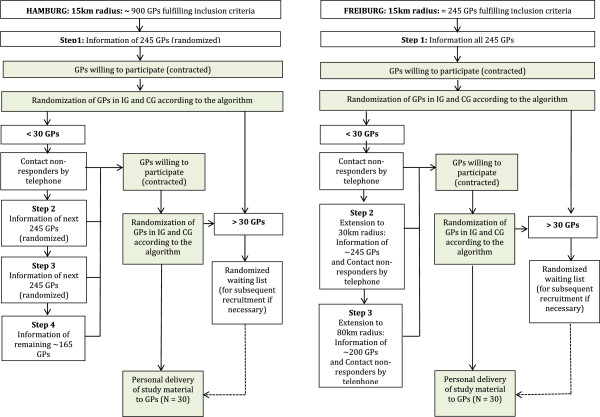
**Stepwise recruitment of GPs in both study centers.** Abbreviations: GP, General Practitioner; IG, Intervention Group; CG, Control Group.

GP offices within a defined radius around the Freiburg and Hamburg city centers are eligible for enrollment at cluster level. Participating offices must further be listed as members of the Association of Statutory Health Insurance Physicians in the state of Baden-Württemberg or Hamburg, respectively. Specialized GPs who do not provide full primary care service, small offices with less than 400 patients per quarter, GPs offering psychotherapy, and GPs taking part in another depression trial are excluded from participation. Both study centers will recruit GPs stepwise until the targeted number of 30 GPs in each center is reached (see Figure 2).

#### Randomization

A cluster-randomized design was chosen over individual patient randomization in order to avoid intervention contamination and logistic complications within the GP offices. Participating GP offices are randomly assigned to either the intervention or the control arm. To prevent foreknowledge of treatment assignment, the allocation of the practices to the study arms will be concealed from research staff, GPs, and CMs until the intervention process is activated in the intervention arm: after obtaining written consent from participating GPs, a research assistant generates a pseudonymized code for each practice and submits this code to an independent statistician at the Department of Medical Psychology in Hamburg. Participating practices are then assigned to the intervention or control group with a 1:1 ratio according to a computer-generated randomization schedule prepared by the statistician (STATA 12.1, procedure ralloc). Block randomization is used with three variable block sizes, with randomization stratified by site. After assignment to the intervention or control group, the pseudonymized practice identifiers and the study arm allocation are returned to the research assistant at the respective study site. This information is then used to enable intervention activation at practices in the intervention arm. The randomization schedule for the two sites, including the pseudonymized codes for the practices and their assignment, remains with the statistician. Study objectives, intervention, and data assessment pertain to the individual patient level.

#### Recruitment of patients

Every participating GP is asked to screen his or her patients according to set inclusion and exclusion criteria and to include five patients for trial participation in the GP practice. Patients of the participating GPs who are 60 years of age or older and show moderate depressive symptoms in the scope of a diagnosed depressive episode, recurrent depressive disorder, or dysthymia (International Classification of Diseases, ICD-10, assessment) are eligible for enrollment at the individual level. Symptom severity is measured with the nine-item depression scale of the Patient Health Questionnaire (PHQ-9; inclusion score 10 to 14). Participants must be willing and able to be contacted regularly by the CM via telephone and to participate in a written survey.

For safety reasons and to avoid data contamination due to comorbidity effects, patients meeting any of the following exclusion criteria are excluded from study participation: alcohol or drug abuse, severe cognitive impairment (e.g., dementia), bipolar disorder, psychotic disorder or severe behavioral symptoms, obsessive compulsive disorder, suicidal ideation and other warning signs of suicide as well as active non-pharmacological (or combined) depression treatment by a specialist (e.g., a psychiatrist or psychotherapist) at time of inclusion. Before GPs apply the PHQ-9 questionnaire, potential participants receive oral and written information about the study. If patients agree to participate in the study, both GP and patient sign the informed consent sheet. Patients, GPs, and the corresponding local study center receive a copy of the signed documents. Subsequently, the local study center will send the baseline questionnaire to the patient. Completed questionnaires will be reviewed by the data management team at the local study centers with regard to exclusion criteria, plausibility of the answers, and completeness of the data.

#### Strategy to guarantee the targeted sample size

In case of insufficient patient enrollment by the GPs, local study centers will recruit additional GPs who will be randomly allocated to one of the two study arms by the independent statistician (see above). Patients in the intervention group at each site will receive treatment as specified in the GermanIMPACT treatment plan manual. Patients in the control group at each site will receive treatment as usual (as agreed on with their GP without involvement of a CM and MHS). As the collaborative care approach was developed to support the treatment of depression in older adults in general practices, the comparison to a treatment-as-usual group was chosen to examine if ancillary care has an additional effect on symptom remission. This approach is analogous to the IMPACT study developed in the United States; since GermanIMPACT is an adaption of the original study, the same comparative conditions were chosen.

### Blinding

Since there is no placebo condition, blinding after assignment is impossible. However, patients in the intervention and control groups only receive information pertaining to their respective study arm so that each group is unaware of the condition of the other group. In both groups, patient outcomes are collected by the data management team (assessors) to prevent the involvement of the CM in patient assessment. The assessors will contact patients in both study arms repeatedly to ensure a low rate of missing data. Due to the pseudonymization process, the statistician will, at all times, be unaware of the identity of the participating practices. Moreover, the statistician will analyze the primary and secondary outcome data without knowledge of the subjects’ allocation to the study arms. For additional analyses of the treatment process, this kind of blinding is not possible.

### Intervention

#### Warm hand-off

Upon inclusion, patients in the intervention group schedule an appointment with their GP and their assigned CM at the GP’s office. The familiarity of the setting reduces access barriers to mental health services and increases patient compliance. It is crucial for the intervention process that the CM be personally introduced to the patient by the GP as a member of the treatment triad; the integration of the CM in the treatment plan should not be perceived as an external referral.

#### Care manager-patient contact

In a 60-minute face-to-face session, the CM provides a short overview of the 12-month treatment period and introduces the patient to the basic intervention techniques and the patient workbook. A semi-structured interview serves to assess the patient’s current health status and behavior, symptoms, medication, and psychosocial stressors and resources; the answers serve as a starting point for the individual intervention plan. As a first task, the patient is encouraged to keep an activity journal for the next seven days.

One week later, the initial session is followed by a 60-minute telephone session during which behavioral activation is revisited and patients are sensitized to the positive relationship between activity and mood improvement. With the help of the activity journal, a list of pleasant activities is created from which the patient is encouraged to choose two and to integrate them into their daily routine. After this second session, 30-minute telephone sessions are held every other week. Every session consists of a short symptom assessment via the PHQ-9 and a short interview including questions about GP visits, medication effects and adherence, and experiences with the behavioral activation tasks. Potential barriers for activity planning and realization are carefully analyzed and new tasks for the next 14-day period are selected accordingly. Due to the regular contact between the CM and patient, the CM can monitor and record deviances from the planned intervention and react instantly to difficulties regarding adherence to the protocol.

**Figure 3:**
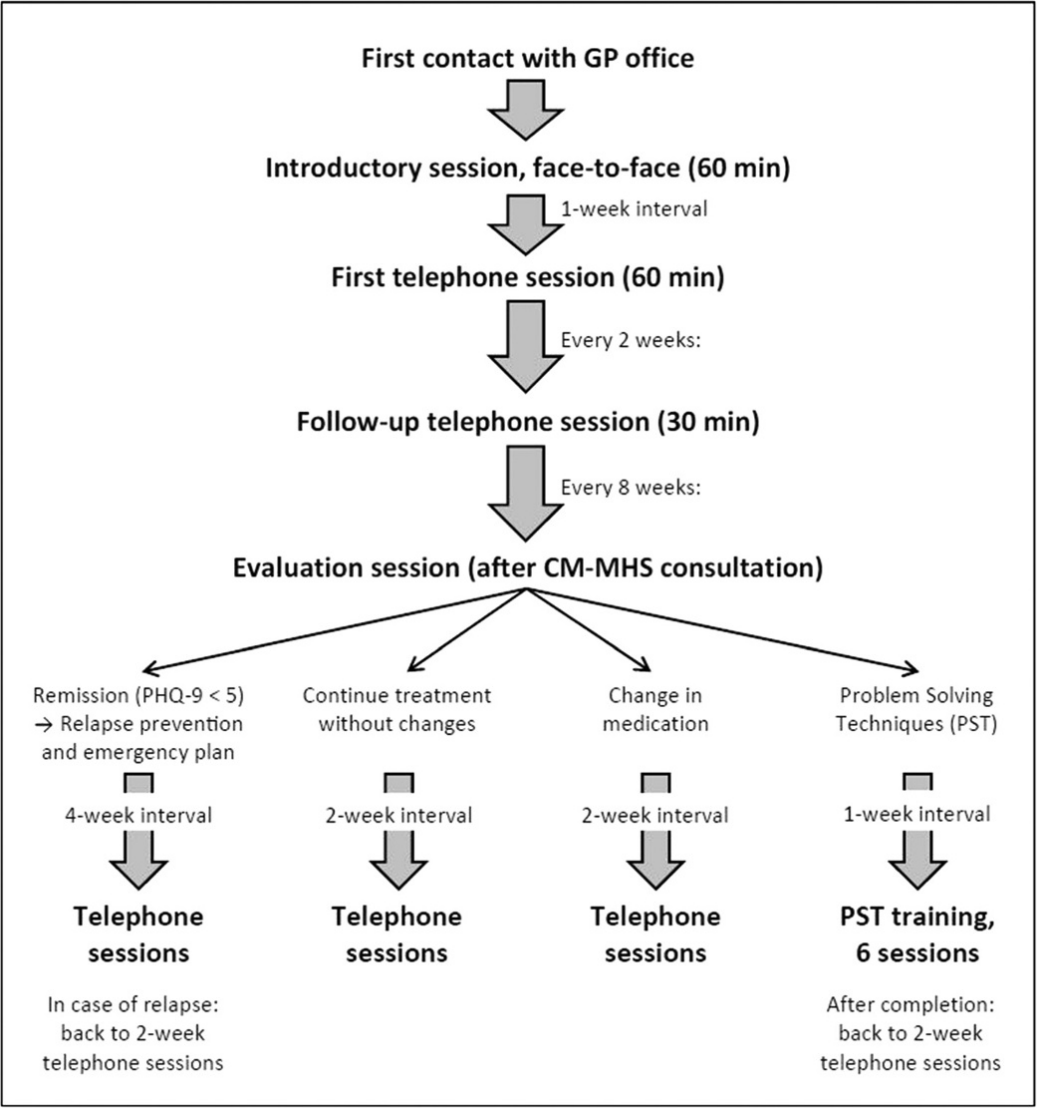
**Sequence of intervention sessions provided by the care manager (total intervention period = 12 months).**

The PHQ-9 questionnaire is used throughout the intervention to assess the current depressive status and to monitor treatment progress. Research shows that administering the PHQ-9 over the phone leads to similar results as an in-person assessment or self-administration [34]. Every eight weeks, a treatment evaluation session is held. Depending on symptom development, the CM can continue the intervention without changes or offer the patient different options for treatment adaptation according to a stepped-care algorithm. In addition to medication-related changes (for example, changes in medication dosage) initiated by the GP and the continuance of activity planning, the CM can provide training sessions focused on problem-solving techniques. This is a brief behavioral intervention intended to reduce depressive symptoms by teaching patients how to systematically solve psychosocial problems. For details on this specific intervention, see Hegel *et al.*[35]. Figure 3 summarizes the 12-month intervention process.

#### Stepped care

Participating GPs are encouraged to start or continue the treatment of depression according to clinical guidelines, including the prescription of antidepressants. They can contact the supervising MHS at any time for consultation.

The continuous assessment of depressive symptoms by the CM allows for the close monitoring of treatment results. CMs and supervising MHSs meet regularly to discuss every patient’s status. Every eight weeks, the need for treatment changes is carefully evaluated. The patient’s PHQ-9 score at baseline serves as the reference parameter for symptom changes at the eight-week intervals. Recommendations for treatment adaptation are based on a stepped-care algorithm as displayed in Table 1. The stepped-care approach allows for modifications of the intervention for each individual participant according to his or her current health status. The criteria for choosing the best option within the possibilities of the stepped-care algorithm are the professional assessment of the GP and the MHS as well as the personal preference of the patient.

**Table 1:** **GermanIMPACT stepped-care algorithm**

Change of current PHQ score compared to baseline	Recommended step
**Improvement of less than 50%** (including no improvement and worsening of symptoms)	a) Medication-related changes (initiated by the GP) including initiation if patient is currently without medication, or
	b) Training of problem-solving techniques provided by the care manager: two face-to-face sessions, four telephone sessions (approximately 45 minutes each); afterwards resumption of telephone sessions at two-week intervals
**Improvement of 50% or more**	No treatment changes
**PHQ-9 score <5** (remission)	c) Discussion of a relapse prevention and emergency plan
	d) Reduction of telephone sessions to four-week intervals; in case of relapse, resumption of telephone sessions at two-week intervals

### Outcome measures and assessment

#### Primary outcome

Primary outcome is the change of the PHQ-9 score [36] as a measure of depressive symptoms over the course of the study from baseline to the end of intervention. In line with similar studies [19, 25, 31], response to treatment is defined as a symptom reduction of 50% or more, and remission is defined as a PHQ-9 score below 5. The PHQ-9 is considered a valid instrument for subgroups of primary care patients with a high prevalence of major depressive episodes [37].

#### Secondary outcomes

Cost-effectiveness measures include the EQ-5D quality of life questionnaire [38] and a German questionnaire on resource utilization (Fragebogen zur Inanspruchnahme medizinischer und nicht-medizinischer Versorgungsleistungen im Alter, FIMA) [39]. As additional measures, a modified version of the Comorbidity Disease Index (CDI) [40], the seven-item Generalized Anxiety Disorder Scale (GAD-7) [41], and a modified version of the Graded Chronic Pain Scale (CPG) [42] are used. Data on resilience (13-item Resilience Scale RS-13) [43], depression-related behavior [44], problem-solving skills (C Bleich and B Watzke, unpublished manuscript, 2012), and current life situation (for example, housing and hobbies) are also collected. In addition, patients in the intervention group receive a questionnaire on therapy preference (SG Riedel-Heller, unpublished manuscript, 2012, currently not publicly accessible) and program evaluation (own instrument). All instruments were chosen according to high standards of psychometric properties, if available [39, 42, 43, 45].

#### Assessment

Data are collected at baseline (t_0_), after 6 months (t_1_), and after 12 months (t_2_ = end of intervention, primary time of assessment). Patients in the intervention and control group will receive pseudonymous questionnaires by mail and are asked to complete and return them to the GermanIMPACT data management team. Table 2 shows an overview of the outcome measures, instruments, and times of assessment.

**Table 2:** **GermanIMPACT outcome measures, instruments, and times of assessment**

Month	0		6		12*
Time of assessment	t_0_		t_1_		t_2_
**Primary outcome**					
Depression (PHQ-9)	X		X		X
**Secondary outcomes**					
Sociodemographic data	X	partial		partial	
Comorbidity (CDI, modified**)	X		–		X
Anxiety (GAD-7)	X		X		X
Pain (CPG, modified**)	X		X		X
Resource utilization (FIMA)	X		X		X
Preference-based quality of life (EQ-5D)	X		X		X
Resilience (RS-13)	X		X		X
Depression-related behavior (Ludman *et al*. [44], modified**)	X		X		X
Problem-solving skills	X		X		X
Current life situation	X		X		X
**Intervention group only**					
Evaluation of intervention	–		–		X
Therapy preference questionnaire	X		–		–
Depression (PHQ-9) - additional measures	–	each session	–	each session	–

*Primary time of assessment (t_**2**_) after intervention.

**Linguistic and culture-specific modifications

*Abbreviations:* PHQ-9, the Patient Health Questionnaire; CDI, the Comorbidity Disease Index; GAD-7, the seven-item Generalized Anxiety Disorder Scale; CPG, the Graded Chronic Pain Scale; FIMA, Fragebogen zur Inanspruchnahme medizinischer und nicht-medizinischer Versorgungsleistungen im Alter; EQ-5D, EuroQol Group; RS-13, the 13-item Resilience Scale.

To avoid the loss of data at baseline and follow-up assessments, the assessors will contact patients whose questionnaires are missing by phone in intervals of 10 to 14 days. For patients who choose to discontinue the trial or deviate from the intervention protocol, a final telephone survey will be conducted. The follow-up calls by the assessors follow a standard operation procedure (SOP) that was defined to ensure a structured and systematic procedure in terms of subsequent collection of missing data via telephone. This approach is restricted to items that do not relate to the patient’s psychological background, with the exception of the PHQ-9 as primary outcome. If answers to more than one item in the PHQ-9 are missing, the PHQ-9 will be reassessed completely by the data management team (in this case, other data regarding health status will be declared invalid). Any revision has to be signed by the assessor with their name and date. Only completed questionnaires will be passed on to data entry.

According to the SOP, data will be entered corresponding to the defined coding plan in the EpiData Entry Client (Version 1.4.2; EpiData Manager Version 1.4.2 for data export) [46]. Instead of running a double data entry procedure, the entered data will be completely (100%) checked and, if necessary, corrected by a second person on the data management team. Data export files (Stata Version 12.1 and SPSS Statistics Version 21 [33, 47]) from both study centers will be centrally collected at the study center in Hamburg for statistical analysis.

As after assignment the research stuff is not blinded, no external data monitoring committee is needed, and data concerning patient safety and treatment efficacy can be monitored internally. Similarly, auditing of the trial conduct will be administered by the research staff: the data management teams in Freiburg and Hamburg will monitor the completion of the questionnaires, while the study coordinators will ensure adherence to the intervention protocol.

### Statistical analysis

**Figure 4:**
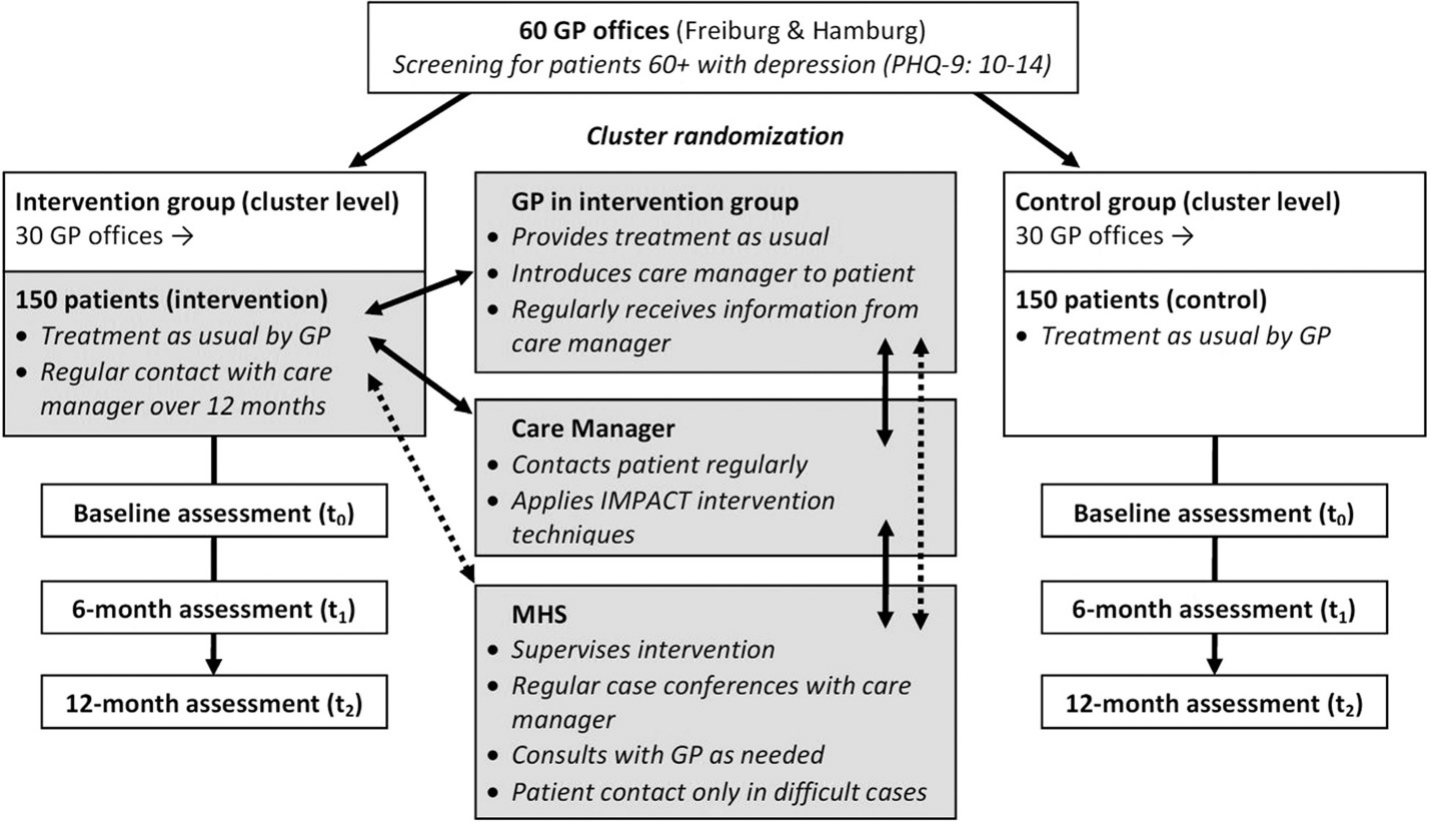
**GermanIMPACT study design.**

The primary analysis will compare the proportion of patients remitted of depressive symptoms at the 12-month follow-up using a mixed-effects logistic regression model in the intention-to-treat sample (including all randomized patients). Treatment condition (intervention versus control) will be treated as a fixed effect. Baseline severity of depressive symptoms will be included as a covariate and variation among patient clusters (treated by the same GPs) will be modeled through random effects. Further binary outcomes will be modeled correspondingly. Continuous outcomes will be analyzed according to the same scheme, but in linear rather than logistic regression models. Item-level missing data in psychometrically sound instruments will be treated in compliance with the corresponding manuals. If no recommendations are available, the expectation-maximization (EM) algorithm will be used to impute up to 30% of missing item responses. Unit-level missing data (patients not providing data for a whole measurement point due to dropping out of the study, for example) will be imputed via the EM algorithm using existing information from the baseline and 6-month follow-up assessments. Further secondary analyses will include analyses in the per-protocol (completer) sample to test the robustness of the primary findings. No interim analysis is planned. Before the start of the analysis, a detailed statistical analysis plan (SAP) will be prepared by the responsible statistician. Intervention-related data are stored and analyzed separately. All analyses will be computed using Stata 12.1. Figure 4 provides a detailed overview of the study design.

### Ethical considerations and safety

#### Good clinical practice

The collaborative care model used in this study has proven beneficial in numerous trials. The treatment plan and intervention techniques are compliant with recommendations of current national and international clinical guidelines. GermanIMPACT follows the regulations of the Federal and State Data Protection Law and the recommendations of the Harmonized Tripartite Guideline for Good Clinical Practice (ICH GCP). All relevant protocol modifications and amendments will be submitted to the responsible ethical review boards and will be reported within the scope of the publication of the trial findings.

Patients with severe depressive symptoms or suicidal ideation at the time of screening are excluded from study participation. GPs are encouraged to refer these patients to an external MHS. Severe depressive symptoms or warning signs of suicide that occur during the intervention can be detected early and be dealt with in a timely manner. In case of adverse advents, such as the emergence of warning signs of suicide in the course of the intervention, the responsible GP and MHS must be consulted instantly by the CM and decide on further action. Different options are possible: 1) GP and MHS can consult to discuss further action; 2) the MHS can make a personal appointment with the patient; or 3) the MHS or GP can initiate further steps, for instance, hospitalization. For patients in the control group, the data management team monitors the development of the depressive symptoms by screening the incoming questionnaires for suicidal cues. A SOP for screening and step-by-step instructions are provided, namely, requesting the patient to see their GP as soon as possible, to make an appointment with a psychiatrist or psychotherapist, or, in case of an emergency, to call emergency medical services directly. The GP and the MHS can then decide about further steps for medical care. All adverse events that occur during the trial will be documented by the CM or, for patients in the control group, by the data management team. Study participation does not exclude the option of referral to an external specialist, although for patients in the intervention group, GPs are encouraged to refer them to the supervising MHS. Thus, both patient groups have full access to all treatment options for depression in the German healthcare system. As treatment by the GP is maintained during and after the trial phase, no post-trial care is intended, and the GP can decide about additional treatment options as needed.

The trial protocol has been granted ethical approval from the ethical review board of the University of Freiburg (approval number 150/12) and the ethical review board of the medical association of Hamburg (approval number MC-224/12). Written informed consent is mandatory for each patient for enrollment in the study.

### Training of GermanIMPACT care providers

The CMs receive comprehensive training by a psychologist familiar with the IMPACT concept and intervention techniques. They are provided with a detailed intervention manual to be used for further study and reference. Regular meetings with the trainers ensure the continuous high standard of the intervention. Clinical concerns are regularly discussed with the supervising MHS. All members of the GermanIMPACT study team are educated and instructed thoroughly in how to deal with emergencies, such as suicidal ideation.

Prior to participation, GPs are individually contacted by members of the study team and informed about the aims of the study and the nature and process of the intervention. Before the inclusion of the first patient, they are personally introduced to the CM assigned to their office.

### Data protection

All patient data collected throughout the study, including documentation of the intervention sessions, are entered into the study database using identifiers (pseudonyms) instead of patient names to grant the highest possible protection of privacy. The necessary exchange of patient data between the providers within the treatment triad is clearly communicated orally, in the patient information and the informed consent form. Health-related and sociodemographic data will be stored separately from personal data. Only authorized staff has access to the data, and data privacy protection according to German Law will be fulfilled. All institutions cooperating in the study are required to agree to the data protection procedures. All investigators will have access to the final trial dataset without any contractual limitations.

#### Dissemination

The trial is registered in the German Clinical Trials Registry (DRKS-ID: DRKS0000358). The results of the trial will be published in international academic journals and national periodicals for healthcare professionals and will be disseminated through presentations at scientific conferences. Participating GPs will receive the publication of the main analyses and additional information in lay language to disseminate them to interested patients. The sponsor will receive a final report including recommendations on development requirements of collaborative care in late-life depression therapy in primary care - according to the results of the trial. There is no restriction on publication. The trial is conducted according to the CONSORT statement, and the study protocol conforms to the Spirit Checklist [48]. The use of professional writers is not intended. So far, no public access to full protocol, participant-level dataset, and statistical code is planned.

## Discussion

The IMPACT approach to treat late-life depression has proven both effective and cost-effective in the United States for more than a decade. Not surprisingly, collaborative models are increasingly recognized in international research on depression care. The adapted GermanIMPACT program is tailored to the specific primary care context in Germany, for instance, with regard to GP medical practice, office organization, infrastructure, communication style, and patient characteristics. By preserving the core principles of the original program, we expect that our collaborative intervention will show effects that are as beneficial as those of its American counterpart.

In the face of the demographic transition and the high prevalence rates of clinically relevant depressive symptoms in the older age groups, collaborative interventions for older adults are a forward-looking approach [2]. We expect that older adults can benefit greatly from a low-threshold intervention like GermanIMPACT that supports, but does not replace the usual treatment by their long-standing family doctor. We hope that the results from our study will help to identify and understand impeding and facilitating factors to the process of integrating CMs into a patient’s treatment plan. Since the notion of collaborative depression care is fairly new to the German healthcare system, we hope that the results of the GermanIMPACT study will yield important insights regarding the applicability of collaborative models and pave the way for the implementation of CMs into outpatient depression treatment in Germany.

## Trial status

Enrollment for the trial began in February 2013. Recruitment is still in progress. Data collection is expected to continue until September 2015.

## Abbreviations

CDC: Collaborative depression care
CDI: the Comorbidity Disease Index
CG: Control Group
CM: Care manager
CPG: the Graded Chronic Pain Scale
EM: expectation-maximization
EQ-5D: EuroQol Group
FIMA: Fragebogen zur Inanspruchnahme medizinischer und nicht-medizinischer Versorgungsleistungen im Alter
GAD-7: the Generalized Anxiety Disorder seven-Item Scale
GCP: Good Clinical Practice
GP: General practitioner
ICD-10: International Classification of Diseases
ICH: International Conference on Harmonization
IG: Intervention Group
IMPACT: Improving Mood Promoting Access to Collaborative Treatment
MHS: Mental health specialist
PHQ-9: the Patient Health Questionnaire
PST: Problem-solving techniques
RS-13: the 13-item Resilience Scale
SAP: Statistical analysis plan
SOP: Standard operation procedure.
